# Copy number variations and stroke

**DOI:** 10.1007/s10072-016-2658-y

**Published:** 2016-07-08

**Authors:** Valeria Colaianni, Rosalucia Mazzei, Sebastiano Cavallaro

**Affiliations:** Institute of Neurological Sciences, CNR, National Research Council, Loc.Burga, 87050 Mangone, CS Italy

**Keywords:** Stroke, DNA copy number variants, Comparative genome hybridization (CGH) arrays, Single-nucleotide polymorphism (SNP) arrays

## Abstract

Stroke is the third leading cause of death worldwide after heart disease and all forms of cancers. Monogenic disorders, genetic, and environmental risk factors contribute to damaging cerebral blood vessels and, consequently, cause stroke. Developments in genomic research led to the discovery of numerous copy number variants (CNVs) that have been recently identified as a new tool for understanding the genetic basis of many diseases. This review discusses the current understanding of the types of stroke, the existing knowledge on the involvement of specific CNVs in stroke as well as the limitations of the methods used for detecting CNVs like SNP-microarray. To confirm an unequivocally association between CNVs and stroke and extend the current findings, it would be desirable to use another methodology to detect smaller CNVs or CNVs in genomic regions poorly covered by this technique, for instance, CGH-array.

## Introduction

Stroke is the third leading cause of death worldwide after heart disease and all forms of cancers. Each year about 795,000 people in the USA suffer from a recurrent or new stroke [1]. This pathology is one of the leading contributors to death and long-term adult disability worldwide, and for this reason, the burden of stroke is felt physically, socially, economically, and emotionally by patients, by their relatives and health care services [2]. Stroke is defined as a syndrome characterized by a quick development of clinical signs and loss of cerebral functions, with symptoms lasting for over 24 h or leading to death, with an apparent cause of vascular origin [3].

Conventional and genetic risk factors contribute to damaging a cerebral blood vessel and, consequently, cause stroke [4]. Genetics plays a significant role in the development of this disease. In fact, several monogenic disorders cause stroke, as well as the interaction of multiple genes.

A new form of genetic variation, known as copy number variations (CNVs), has been recently identified as a new tool for understanding the genetic basis of many diseases, including stroke. CNVs are deletions and duplications (loss or gain) of segments of genome [5, 6].

CNV may alter the levels of gene expression, may also disrupt genes or regulation elements, may lead to frameshifts, and may generate new fusion products; all these genetic variations can result in a phenotypic variation, susceptibility of an individual to disease and/or a differentiated drug response [7, 8].

Today, modern high-resolution technologies, such as comparative genome hybridization (CGH) arrays, allow to detect simultaneously CNVs in multiple loci. These technologies may be clinically used to identify people who may be at risk for a stroke or might create benefit to identify specific therapies.

This review aims to provide a comprehensive overview of stroke types and their etiopathogenesis and summarize the current knowledge regarding the involvement of CNVs in stroke.

## Stroke types

Occluded or ruptured cerebral blood vessel determines a reduction in normal cerebral blood flow in the affected vascular territory, resulting in reduced nutrient delivery to gray and white matter [9]. Without oxygen and nutrients from blood, neurons start to die within a few minutes in the core of the infarcted area. The region around the core, called “the ischemic penumbra,” contains functionally impaired cells but still viable for the presence of collateral vessels. This area may become infarcted at later time points due to secondary neuronal damage caused by the cascade of biochemical events that occurs after ischemia. This mechanism is common to all types of stroke: ischemic stroke (IS) [2], hemorrhagic stroke (HS), and transient ischemic attack (TIA).

IS represents up to 80 % of all stroke cases reported in epidemiological studies [2]. It is more often disabling rather than fatal, representing the most common life threatening neurological disorder. The remaining 20 % of stroke cases are caused by primary intracerebral hemorrhage (about 15 %) and subarachnoid hemorrhage (about 5 %) with a potential mortality rate from 30 to 50 % within 30 days [10, 11]. Last, TIA is similar to IS but differs in duration (less than 24 h).

This distinction in different stroke types is critical for therapeutic decision, although it is likely that these forms of stroke have both similar and different genetic susceptibility, risk factors, and etiologic overlaps. Furthermore, the patients’ global risk factor profile at the time of the stroke may influence the form of stroke that occurs.

## Risk factors

**Fig. 1:**
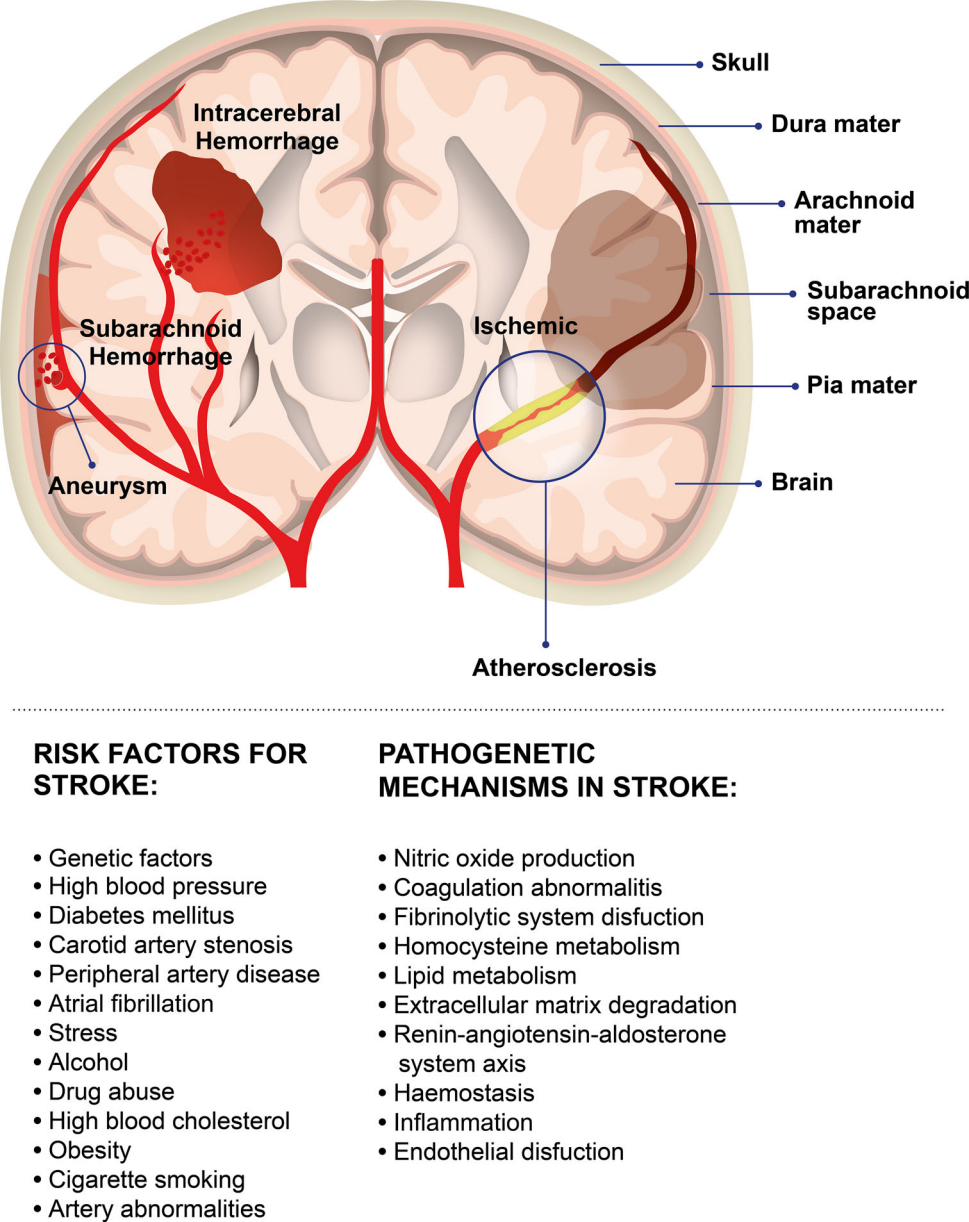
Several pathogenetic mechanisms and a wide variety of risk factors can be correlated with stroke onset such as those indicated in the image

Stroke is a challenging disease to study, because it can depend on a wide variety of risk factors. Conventional risk factors that increase a person’s likelihood of having a stroke can be controllable and uncontrollable (Fig. 1) [12].

Controllable risk factors, by medication or lifestyle changes, primarily include high blood pressure, diabetes mellitus, carotid artery stenosis, peripheral artery disease, atrial fibrillation, stress, alcohol, drug abuse, hypercholesterolemia, obesity, smoking, and physical inactivity.

Uncontrollable risk factors mainly comprise age, race (African–Americans have a much higher risk of death from a stroke than Caucasians—http://www.strokeassociation.org), ethnicity, family history, genetic factors, previous stroke or TIA, artery abnormalities, fibromuscular dysplasia, male gender, etc. Genetic factors contributing to onset of stroke have been identified in twin studies and familial aggregation studies [13]. Genetic predisposition to stroke can be categorized either as a single gene disorder or as a polygenic disorder, although the majority of the studies have mainly focused on monogenic forms of stroke. A recent study demonstrates that conventional cardiovascular risk factors, particularly smoking and hypertension, have been associated with an earlier stroke onset, highlighting the interaction between gene and environment, and the clinical importance of careful risk factor control even in patients with monogenic stroke disorders [14].

## Monogenic stroke diseases

The most common monogenic form is CADASIL (cerebral autosomal dominant arteriopathy with subcortical infarcts and leukoencephalopathy) (OMIM 125310). It results from mutations in the gene *NOTCH3*, which encodes a transmembrane receptor. Mutations result in an odd number of cysteine residues within one of the 34 epidermal growth factor (EGF)-like repeats in the extracellular amino-terminal region of the Notch3 receptor, leading to its abnormal accumulation at the cytoplasmic membrane of vascular smooth muscle cells, in the vessels of patients [15].

Recently, genes involved in several other rare monogenic diseases have been recognized. CARASIL (cerebral autosomal recessive arteriopathy with subcortical infarcts and leukoencephalopathy) (OMIM 600142) causes lacunar stroke and early onset vascular dementia, and derives from recessive mutations in the HtrA serine protease (*HTRA1*) gene, which is involved in TGF-beta signaling [16].

*COL4A1* and *COL4A2* are two genes that encode the alpha 1 and alpha 2 chains of type IV collagen, which cause autosomal dominant porencephaly, infantile hemiparesis, and childhood hemorrhage [17, 18].

Another monogenic condition characterized by visual loss, stroke, and dementia is autosomal dominant retinal vasculopathy with cerebral leukodystrophies (RVCL) (OMIM 192315), a microvascular endotheliopathy. Mutations in the *TREX1* gene are responsible for this disease [19].

Mutations in the genes underlying these monogenic forms of stroke are not recognized as the cause for multifactorial stroke, but may help in their comprehension.

## Multifactorial stroke

Single mutations can induce stroke, but in most cases, this condition is caused by interaction among multiple genes. Several candidate pathways have been examined in stroke, including those involved in endothelial function, nitric oxide production, renin–angiotensin–aldosterone system, coagulation, haemostasis, and inflammation (Fig. 1).

Nowadays, because of the completion of Human Genome Project, modern high-throughput technologies, including the next generation sequencing (NGS), CGH, and single-nucleotide polymorphism (SNP) arrays, can be used to genotype simultaneously multiple genes involved in stroke. The subsections below will describe the CNVs and the methods to detect them to assess the potential association between CNVs and the development of stroke.

## CNV

CNVs are defined as deletions or duplications of DNA that produce any change in copy number of a specific chromosomal region [5, 6, 20]. Their size varies from one kilobase (kb) to several megabases (Mb) [21, 22], and they often involve one or more genes [6].

In a diploid cell, the number of copies of a locus is two, a copy inherited from the mother and the other from the father, but some loci may contain CNVs.

It has been estimated that about 12 % of the genome is covered by CNVs and more than 41 % identifies CNVs overlap with known genes [6, 23, 24]. CNVs play an important role in the genome variability allowing humans to evolve and adapt [6, 20, 25].

CNVs have been recognized as source of both normal genetic variation and pathogenic mutation [26]. They can destroy regulation elements, generating new fusion products with various possible positive or negative consequences [20, 27]. Other studies indicate that larger CNVs are associated with pronounced clinical characteristics and deletions are associated with more severe phenotypes than duplications [6, 7].

CNVs may be divided into inherited or de novo types, and this depends on whether they are transmitted or not by at least one parent [28].

CNVs are classified into different categories (Table 1). Common CNVs usually represent normal genomic variation or benign. Rare CNVs can be likely benign variant specific to an individual or family, pathogenic variant, likely pathogenic variant and variant of unknown significance (VOUS)–CNVs with uncertain clinical and functional relevance. VOUS occur when a new CNV is identified. Family studies may help clinical interpretation, because the presence of a de novo CNV that segregates with the pathological phenotype strengthens the evidence that it is pathogenic. However, the importance of some CNVs may be still uncertain even after studies on families because of their variable expressivity; for this reason, it is extremely useful to perform comparative case–control data analysis in large populations to definitively associate specific CNVs to human diseases. Some CNVs do not lead to phenotypic effect in the carrier, but they can create genomic instability in future generations [20]. Normal genomic variants or benign CNVs may sometimes indirectly cause or contribute to pathogenicity, for instance, if:

**Table 1:** CNV classification in human genome

Type CNV	CNV classification
Common	Benign CNV
Rare	Likely benign CNV
	CNV of uncertain clinical relevance
	CNV of possible clinical relevance
	CNV of clinical relevance

- each parent takes the same heterozygous deletion on an allele, hence, two benign heterozygous deletions generating a deleterious homozygous deletion;
- each parent has a different, benign heterozygous deletion in the same gene, when both parental mutations are inherited, they cause a deleterious effect in the offspring;
- CNV on the X chromosome in an unaffected mother can be deleterious when inherited by a son [29, 30];
- there is a deletion on one allele and a mutated gene on the other allele [31];
- the CNV occurs in combination with another CNV and this leads to a pathogenic effect [32].

For all these reasons, a better understanding of all mechanisms underlying CNVs is required.

## Methods to detect CNVs

Different methods for detecting CNVs are available, including real-time PCR (RT-PCR), NGS, and microarrays. The last one is now the primary method used for CNVs detection.

Microarrays include both SNP- and CGH-arrays. These technologies allow detection of CNVs at higher resolution than classical cytogenetic methods [5, 33]. The application of CGH- and SNP-arrays in control cohorts produces a genome-wide architecture of CNVs named ‘‘CNV landscape’’ [26, 34].

Array-based technologies have emphasized recurrent CNVs that seem to be associated with some diseases; in effect, they have been identified more frequently in patients compared with control populations.

All these methods differ in their ability to detect deletions or duplications; for instance, more duplications are missed by SNP-array and NGS approaches than by CGH-array. Currently, CGH-array is the most sensitive tool for the research of small differences in CNVs [35].

**Fig. 2:**
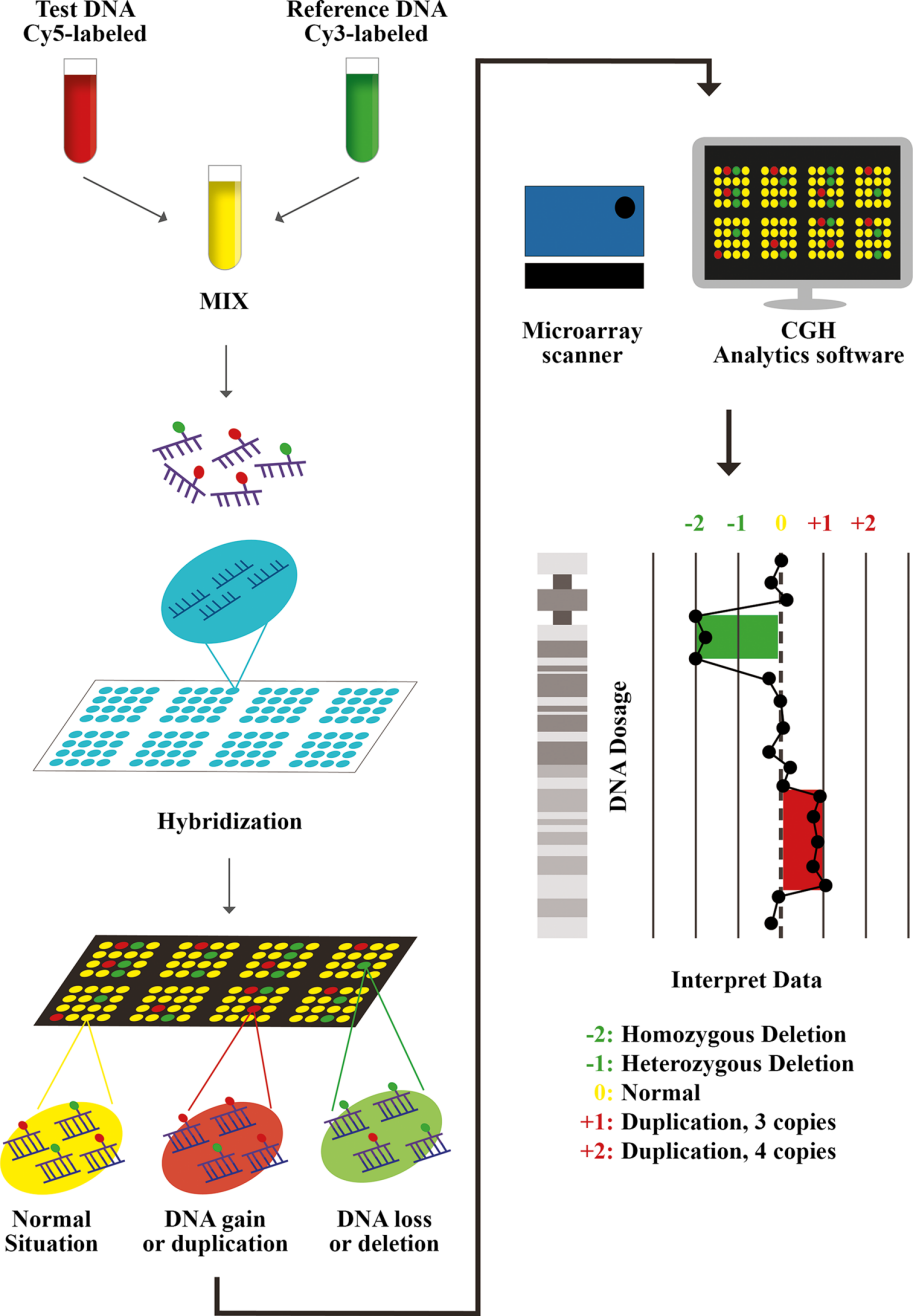
Array CGH procedure is characterized by the isolation of DNA from a patient/test and from a control/reference, independent labeled with two different fluorophores of different colors (usually *red-cyanine* 5 and *green-cyanine* 3), and consequently, hybridized on array containing thousands of known probes. The probes are arranged in a precise grid on chip. The microarray scanner detects the fluorescent signals on each probe. Last, array analytical software calculates the log_2_ ratio of fluorescence (Cy5/Cy3), and in this way, deletions or duplications in DNA can be identified. A higher intensity of the test sample color in a specific region of a chromosome versus the control indicates the gain of DNA of that region, while a higher intensity of the control sample color versus the test sample indicates the loss of material in that specific region. A neutral color (*yellow* when are used *red* and *green* fluorophores) indicates no difference between the two samples in that location so a normal condition

CGH-array allows to detect chromosome imbalances too tiny to be seen with the microscope. DNA samples from a patient and from a control are labeled with two different fluorophores and, consequently, hybridized on array containing thousands of known DNA probes. The probes are arranged in a precise grid on a glass slide called “chip” [36, 37]. The most commonly used fluorochromes are red and green (cyanine 5 and cyanine 3). The chip is analyzed in a microarray scanner which measures the amount of red and green fluorescence on each probe. Last, an array analytical software calculates the ratio of fluorescence and in this way deletions or duplications in DNA can be identified (Fig. 2).

## CNVs in ischemic stroke

CNVs are associated with several complex disorders, and their potential association with risk for stroke has been object of lively discussion [38].

Until now, there are a relatively few association studies between CNVs and patients with IS.

In the first, genome-wide analysis was investigated whether CNVs could modulate risk for IS and was intended to provide a list of CNVs in IS patients, but no common genomic structural variation unequivocally linked to IS was detected [39]. CNVs were examined in 263 patients with IS and 275 neurologically normal controls using SNP chips (Illumina Inc., CA, USA). In 146 patients, the authors identified a total of 231 CNVs resulting in simple deletions or duplications. Most of the same CNVs were identified in healthy individuals too. Forty-five CNVs (19.5 %) were unique (Table 2). Within these new potential sites of structural variation, only one genomic region, on chromosome 1, contained recurrent CNVs in three individuals with IS. These individuals showed an apparently identical duplication spanning the genes SPRY domain-containing SOCS box protein 1 (*SPSB1*) and hexose-6-phosphate dehydrogenase (*H6PD*). Because of the potential clinical relevance of these alterations, they examined copy number at this locus in an additional 450 neurologically normal samples. These data showed the presence of CNVs at this locus in five of these samples (~1 %), suggesting that these variants were not a risk factor for IS. The remaining CNVs could have a role in the pathobiology of IS; however, due the low frequency of each individual alteration, screening of these variants in a greater cohort would be required to confirm the association unequivocally. In addition, it would be desirable to use another methodology to detect smaller CNVs or CNVs in genomic regions poorly covered by this technique that could confirm the risk for IS.

**Table 2:** CNVs in stroke

Type of stroke	Position (chr: start–end)	Size (kb)	CN-state	Genes	References
IS	1:9243800–9309900	66,1	Gain	H6PD, SPSB1	Matarin et al. [35]
	1:9246500–9335000	88,5	Gain	H6PD, SPSB1	
	1:9246500–9336000	89,5	Gain	H6PD, SPSB1	
	1:173728000–173984000	256	Gain		
	1:226872000–226995000	123	Gain	FTHL2, RHOU	
	3:163361400–163421900	60,5	Gain		
	3:184938000–185228000	290	Gain	YEATS2,MAP6D1, PARL, LOC391598, LOC647265	
	3:101837000–101916300	79,3	Gain	GPR128, TFG	
	4:14308000–14516000	208	Gain		
	4:100903800–100966200	62,4	Gain	DAPP1	
	4:81604000–82138000	534	Loss	C4orf22	
	5:75963000–76129000	166	Gain	IQGAP2, F2R	
	5:120960000–121059000	99	Gain		
	6:62042000–62094400	52,4	Gain		
	6:96663300–96713300	50	Gain	FUT9	
	6:124750188–124907081	156,893	Gain	TCBA1	
	6:161565000–161770000	205	Gain	AGPAT4, PARK2	
	6:113575000–114025000	450,000	Loss	LOC643884, LOC728590	
	7:8174000–8470000	296	Gain	ICA1, NXPH1	
	7:122818668–123545119	726,451	Gain	FLJ35834, NDUFA5, ASB15, LOC442721, WASL, HYALP1, HYAL4, SPAM1, LOC730130	
	8:1082000–1295000	213	Gain		
	8:25511200–25543900	32,7	Gain		
	8:43260000–43911000	651	Gain	POTE8, LOC728563	
	9:16949000–17061000	112	Loss		
	9:17588300–17623200	34,9	Gain	SH3GL2	
	9:9465000–9563000	98	Loss		
	10:25999000–26066800	67,8	Gain or triplication		
	11:39007000–39120000	113	Gain		
	11:107262000–107444000	182	Gain	CUL5, RAB39, LOC643949	
	13:67676000–67798000	122	Gain		
	13:54036000–54422000	386	Loss		
	13:85599004–85842380	243,376	Loss		
	15:53302000–53546000	244	Gain	RAB27A, PIGB, CGPG1, MIRN628, DYX1C1, LOC729120	
	15:83799700–83875900	76,2	Gain or triplication	AKAP13	
	15:88651000–88800000	149	Gain	GABARAPL3, MGC75360, IQGAP1	
	18:7803000–8013000	210	Gain	PTPRM	
	18:72553700–72598400	44,7	Gain		
	19:61175000–61284000	109	Gain	NALP8, NALP5, LOC729982	
	19:62695000–62888000	193	Gain	ZNF419, MGC4728, ZNF549, ZNF550, ZNF416, ZIK1, ZNF530, ZNF134, ZNF211, ZSCAN4, ZNF551	
	20:51262600–51307100	44,5	Gain	TSHZ2	
	21:34417000–36526000	2,109	Gain	LOC728778, LOC728556, RP9P1, CBR3, DOPEY2, RPL3P1	
	X:75202600–75274600	72	Gain		
IS			Loss	GSTM1, GSTT1	Nørskov et al. [36]
IS					Grond-Ginsbach et al. [40]
CeAD-associated Cnvs detected in 49 patients EM+					
	18:59640388–59694035	54	Loss	SERPINB2	
	4:144570037–144634141	64	Gain	GAB1	
	12:91609585–91681005	71	Loss	C12orf74, PLEKHG7	
	9:107430028–107517525	87	Loss	FKTN, TAL2, TMEM38B	
	6:161612276–161734297	122	Gain	AGPAT4, PARK2	
	3:113368966–113538579	170	Loss	SLC9A10, CD200	
	19:52912535–53094035	182	Gain	GLTSCR1, EHD2, GLTSCR2, SEPW1, TPRX1, CRX, SULT2A1	
	6:183515–671736	488	Gain	EXOC2, IRF4, DUSP22, HUS1B, AL031770	
	1:202619544–203219581	600	Gain	PPP1R15B, PIK3C2B, MDM4, LRRN2, NFASC	
	2:189109859–189763802	654	Loss	GULP1, DIRC1, COL3A1, COL5A2	
	19:1971710–2632482	661	Gain	MKNK2, C19orf36, MOBKL2A, AP3D1, DOT1L, IZUMO4, AC004410, PLEKHJ1, C19orf35, SF3A2, AMH, JSRP1, OAZ1, LINGO3, AC104537.1, LSM7, TIMM1, TMPRSS9, LMNB2, GADD45B, GNG7, DIRAS1, SLC39A3, SGTA	
	20:31395708–32782473	1387	Loss	CDK5RAP1, SNTA1, CBFA2T2, NECAB3, C20orf144, PXMP4, C20orf134, E2F1, ZNF341, RALY, EIF2S2, ASIP, AHCY, ITCH, DYNLRB1,MAP1LC3A, PIGU, TP53INP2, NCOA6	
	4:141190354–144311522	3121	Gain	SCOC, CLGN, ELMOD2, TBC1D9, RNF150, ZNF330, IL15, INPP4B	
CeAD-associated CNVs detected in 21 patients with EM−					
	8:14006431–14131717 125	125	Loss	SGCZ	
	7:132844963–132988175	143	Loss	EXOC4	
	10:68972491–69137046	165	Gain	CTNNA3	
	16:11935326–12115916	181	Gain	RP11-166B2.1, TNFRSF17, RUNDC2A, SNX29, AC00760.1	
	2:133324676–133563950	239	Loss	NCKAP5	
IS	Exons 35–52		Duplication	VWF	Nik-Zainal et al. [41]
SAH	rs1242541		Loss	SEL1L	Bae et al. [42]
SAH	4:153210505–153212191	1.7	Loss	PET112 L, FBXW7	Bae et al. [43]
	10:6265006–6267388	2.4	Gain	RBM17, PFKFB3	

*CeAD* cervical artery dissection, *EM+* patients with electron microscopic alterations, *EM* patients without electron microscopic alterations, *IS* ischemic stroke, *SAH* subarachnoid hemorrhage

Nørskov and colleagues evaluated whether CNVs in glutathione S-transferases (*GSTs*) *M1* and *T1* genes were associated with an increased risk of ischemic vascular disease (IVD) including IS [40]. GSTM1 and GSTT1 detoxify the products of oxidative stress and may protect against atherosclerosis and IVD. Furthermore, epidemiological studies hypothesized that CNVs in *GSTM1* and *GSTT1* genes were associated with progressive decreases in their catalytic activity. In addition, they may modify risk of atherosclerosis and increase risk of IS (Table 2) [41]. The researchers included 6.557 IVD cases and 16.502 controls from 2 general population studies and 2 case–control studies. To genotype for the exact number of genes copies of *GSTM1* and *GSTT1*, they used the RT-PCR. Principal findings in these studies individually or combined demonstrated CNVs in *GSTM1* and *GSTT1* were not associated with the risk of IS or any ischemic vascular event. Furthermore, the authors did not detect any associations between smoking exposure and *GSTM1* and *GSTT1* genotype (Table 2).

IS can also be caused by spontaneous cervical artery dissection (CeAD), in particular in healthy young adults [42, 43], but unfortunately the etiology of CeAD is still unknown. A genetic predisposition seems to be associated between CeAD and inherited microscopic and submicroscopic connective tissue alterations. Grond-Ginsbach and collaborators searched for causative CNVs in patients with and without connective tissue alterations that may predispose to CeAD [44]. They included 49 non-traumatic CeAD-patients with electron microscopic alterations (EM+ patients), 21 patients without alterations (EM− patients), and 403 control subjects. All patients were screened for CNVs through Affymetrix SNP6.0 microarrays. The authors concluded that rare genetic variants may contribute to the pathogenesis of CeAD in particular in EM+ patients (Table 2). However, the risk for CeAD might not be related to a single-gene or a single-genetic pathway, but it might be associated with different genetic variants (Table 2).

Nik-Zainal and colleagues examined a case of a 35-year-old male with a ring chromosome 12 originally diagnosed 20 years before IS appeared [45]. CGH-array analysis revealed a submicroscopic microdeletion and microduplication within 12p13.3 and a microdeletion in 12q24.33. FISH analysis further revealed that in this patient, the duplication from exons 35–52 of Von Willebrand factor (*VWF*) gene was in an inverted orientation within the ring chromosome. VWF plays a critical role in maintaining the normal balance of the clotting cascade via multiple complex interactions with factor VIII, platelets, collagen, and subsequent degradation by a metalloprotease called ADAMTS13 (A Disintegrin And Metalloprotease with ThromboSpondin Type-1 Motif, 13). Partial duplication of this gene suggests that a potential mechanism for generating a prothrombotic state may have contributed to a premature stroke (Table 2).

## CNVs in hemorrhagic stroke

At present, only two studies from the same authors reported the relationship between CNVs and HS, in particular with subarachnoid hemorrhage (SAH) (Table 2).

In 2008, Bae and colleagues genotyped SNPs on CNV regions for the CNVs identification. They found out 597 SNP markers with a multiallelic CNV genotype, known as the common deletion polymorphism, within the CNV region. Among 597 CNV markers, CNV region around rs1242541 (nearest gene: *SEL1L*) showed the most significant association with the risk of SAH [46].

In 2010, they executed the first genome-wide association study to investigate the relationship between common CNVs and SAH. They hypothesized that CNVs can predict the risk of SAH [47]. The authors identified a total of 4.574 CNVs from a Japanese population sample (*n* = 473) and discovered 1.644 unique CNV regions containing 1.232 genes. The researchers carried out a genome-wide CNV association analysis using a logistic regression model, controlling for age and sex, to determine the association between the identified CNVs and the risk of SAH in 187 CNVs with frequency >1 %. Interestingly, two CNV regions, deletion 4q31.3 and duplication 10p15.1 have been significantly associated with the risk of SAH. In the case of chr4:153210505–153212191, the frequency of deletion in the patients group was higher than that in the control group. This result suggests that the deletion allele may be a risk factor for SAH. In the case of chr10:6265006–6267388, the frequency of duplication in the patients group was higher than that in the control group. This latter finding indicates that the increase in copy number in the region may influence the onset of SAH. Unlike their previous work, in this study, no significant association has been detected between CNV region around rs1242541 and the risk of SAH. Probably, these discrepancies in the results may be due to the fact that this last study was conducted at a larger scale.

Finally, investigations on the association between CNVs and intracerebral hemorrhage (ICH) have never been reported in the literature.

## Discussion

Several factors may increase the risk of stroke, including genetic ones, and in particular, CNVs. Even if the role of CNVs in the genetic etiology of stroke is not yet well established, there is an increasing interest in CNVs because of their usefulness as a powerful tool in understanding the genetic basis of numerous diseases.

Until now, there are a relatively few association studies between CNVs and stroke. Some studies concluded that rare CNVs may contribute to the pathogenesis of stroke, while other studies detected no significant association between specific CNVs and the risk of stroke. These seemingly contradictory data can arise, because in most studies, the method used for detecting CNVs was SNP-microarray. To confirm an unequivocally association between CNVs and stroke and extend the current findings, it would be desirable to use another methodology to detect smaller CNVs or CNVs in genomic regions poorly covered by this technique, for instance, CGH-array. In addition, more duplications are missed by SNP-based array and sequencing than by CGH-array and SNP-array, which have limited ability to detect single-exon CNVs due to the distribution of SNPs across the genome. Currently, CGH-array is the most sensitive tool for the research of CNVs. Another strategy to improve the detecting of CNVs would be that to combine SNP-microarray and CGH-array into one platform providing a genetic screening in a more efficient manner.

Furthermore, screening of CNVs in a suitable number of patients would be required to confirm unambiguously the association between CNVs and risk for stroke.

It is clear that the discovery of disease-associated CNVs will lead to improvements in clinical genetic diagnosis and genetic counseling. This will not only help to make more appropriate diagnosis but may help to design treatments which could be allocated according to genetic etiology rather than meeting strict diagnostic criteria set for each separate disorder. Therefore, the identification of CNVs could lead to personalized medical treatments which would be targeted for each patient and his genome, and could, therefore, improve treatment success.

Finally, as patients with shared genetic etiologies of stroke will be identified, studies of genotype–phenotype correlation, natural history, and therapeutic response to specific drugs can be performed, which will lead to improved long-term care and outcomes for patients.

In light of these observations, further studies will be required to clarify how CNVs may affect an individual’s susceptibility to stroke, to confirm the associations in larger populations, and to know if there are some association between CNVs and the different subtypes of stroke.

## References

[1] Roger VL, Go AS, Lloyd-Jones DM (2011). Heart disease and stroke statistics—2011 update: a report from the American Heart Association. Circulation.

[2] Feigin VL, Lawes CM, Bennett DA, Barker-Collo SL, Parag V (2009). Worldwide stroke incidence and early case fatality reported in 56 population-based studies: a systematic review. Lancet Neurol.

[3] Hatano S (1976). Variability of the diagnosis of stroke by clinical judgement and by a scoring method. Bull World Health Org.

[4] Warlow C, Sudlow C, Dennis M, Wardlaw J, Sandercock P (2003). Stroke. Lancet.

[5] Cook EH, Scherer SW (2008). Copy-number variations associated with neuropsychiatric conditions. Nature.

[6] Redon R, Ishikawa S, Fitch KR (2006). Global variation in copy number in the human genome. Nature.

[7] Freeman JL, Perry GH, Feuk L (2006). Copy number variation: new insights in genome diversity. Genome Res.

[8] Henrichsen CN, Vinckenbosch N, Zöllner S (2009). Segmental copy number variation shapes tissue transcriptomes. Nat Genet.

[9] Dirnagl U, Iadecola C, Moskowitz MA (1999). Pathobiology of ischaemic stroke: an integrated view. Trends Neurosci.

[10] Broderick J, Connolly S, Feldmann E (2007). Guidelines for the management of spontaneous intracerebral hemorrhage in adults. Stroke.

[11] Fogelholm R, Murros K, Rissanen A, Avikainen S (2005). Long term survival after primary intracerebral haemorrhage: a retrospective population based study. J Neurol Neurosurg Psychiatry.

[12] Sacco RL, Ellenberg JH, Mohr JP, Tatemichi TK, Hier DB, Price TR, Wolf PA (1989). Infarcts of undetermined cause: the NINCDS stroke data bank. Ann Neurol.

[13] Cole JW, Meschia JF (2011). Stroke genetics update: 2011. Curr Cardiovasc Risk Rep.

[14] Adib-Samii P, Brice G, Martin RJ, Markus HS (2010). Clinical spectrum of CADASIL and the effect of cardiovascular risk factors on phenotype: study in 200 consecutively recruited individuals. Stroke.

[15] Joutel A, Corpechot C, Ducros A (1996). Notch3 mutations in CADASIL, a hereditary adult-onset condition causing stroke and dementia. Nature.

[16] Hara K, Shiga A, Fukutake T (2009). Association of HTRA1 mutations and familial ischemic cerebral small-vessel disease. N Engl J Med.

[17] Gould DB, Phalan FC, van Mil SE (2006). Role of COL4A1 in small-vessel disease and hemorrhagic stroke. N Engl J Med.

[18] Jeanne M, Labelle-Dumais C, Jorgensen J (2012). COL4A2 mutations impair COL4A1 and COL4A2 secretion and cause hemorrhagic stroke. Am J Hum Genet.

[19] Richards A, van den Maagdenberg AM, Jen JC (2007). C-terminal truncations in human 3′–5′ DNA exonuclease TREX1 cause autosomal dominant retinal vasculopathy with cerebral leukodystrophy. Nat Genet.

[20] Feuk L, Carson AR, Scherer SW (2006). Structural variation in the human genome. Nat Rev Genet.

[21] Sebat J, Lakshmi B, Troge J (2004). Large-scale copy number polymorphism in the human genome. Science.

[22] Buckland PR (2003). Polymorphically duplicated genes: their relevance to phenotypic variation in humans. Ann Med.

[23] Carter NP (2007). Methods and strategies for analyzing copy number variation using DNA microarrays. Nat Genet.

[24] Reichenberg A, Weiser M, Rabinowitz J (2002). A population-based cohort study of premorbid intellectual, language, and behavioral functioning in patients with schizophrenia, schizoaffective disorder, and nonpsychotic bipolar disorder. Am J Psychiatry.

[25] Conrad DF, Pinto D, Redon R (2010). Origins and functional impact of copy number variation in the human genome. Nature.

[26] Díaz de Ståhl T, Sandgren J, Piotrowski A (2008). Profiling of copy number variations (CNVs) in healthy individuals from three ethnic groups using a human genome 32 KBAC-clone-based array. Hum Mutat.

[27] Lupski JR, Stankiewicz P (2005). Genomic disorders: molecular mechanisms for rearrangements and conveyed phenotypes. PLoS Genet.

[28] McCarroll SA (2008). Extending genome-wide association studies to copy-number variation. Hum Mol Genet.

[29] De Leeuw N, Bulk S, Green A, Jaeckle-Santos L (2010). UBE2A deficiency syndrome: mild to severe intellectual disability accompanied by seizures, absent speech, urogenital, and skin anomalies in male patients. Am J Med Genet A.

[30] Ramocki MB, Tavyev YJ, Peters SU (2010). The MECP2 duplication syndrome. Am J Med Genet A.

[31] Zhang H, Gao J, Ye J, Gong Z, Gu X (2011) Maternal origin of a de novo microdeletion spanning the ERCC6 gene in a classic form of the Cockayne syndrome. Eur J Med Genet 54 10.1016/j.ejmg.2011.03.012 21477668

[32] Girirajan S, Rosenfeld JA, Cooper GM (2010). A recurrent 16p12.1 microdeletion supports a two-hit model for severe developmental delay. Nat Genet.

[33] Slavotinek AM (2008). Novel microdeletion syndromes detected by chromosome microarrays. Hum Genet.

[34] Zogopoulos G, Ha KC, Naqib F (2007). Germ-line DNA copy number variation frequencies in a large North American population. Hum Genet.

[35] Pinto D, Darvishi K, Shi X (2011). Comprehensive assessment of array-based platforms and calling algorithms for detection of copy number variants. Nat Biotechnol.

[36] Solinas-Toldo S, Lampel S, Stilgenbauer S (1997). Matrix-based comparative genomic hybridization: biochips to screen for genomic imbalances. Genes Chromosomes Cancer.

[37] Pinkel D, Segraves R, Sudar D (1998). High resolution analysis of DNA copy number variation using comparative genomic hybridization to microarrays. Nat Genet.

[38] Lupski JR (2007). Structural variation in the human genome. N Engl J Med.

[39] Matarin M, Simon-Sanchez J, Fung HC (2008). Structural genomic variation in ischemic stroke. Neurogenetics.

[40] Nørskov MS, Frikke-Schmidt R, Loft S, Sillesen H, Grande P, Nordestgaard BG, Tybjaerg-Hansen A (2011). Copy number variation in glutathione S-transferases M1 and T1 and ischemic vascular disease four studies and meta-analyses. Circ Cardiovasc Genet.

[41] Stephens JW, Bain SC, Humphries SE (2008). Gene-environment interaction and oxidative stress in cardiovascular disease. Atherosclerosis.

[42] Schievink WI (2001). Spontaneous dissection of the carotid and vertebral arteries. N Engl J Med.

[43] Debette S, Leys D (2009). Cervical-artery dissections: predisposing factors, diagnosis, and outcome. Lancet Neurol.

[44] Grond-Ginsbach C, Chen B, Pjontek R (2012). Copy number variation in patients with cervical artery dissection. Eur J Hum Genet.

[45] Nik-Zainal S, Cotter PE, Willatt LR, Abbott K, O’Brien EW (2011). Ring chromosome 12 with inverted microduplication of 12p13.3 involving the Von Willebrand Factor gene associated with cryptogenic stroke in a young adult male. Eur J Med Genet.

[46] Bae JS, Cheong HS, Kim JO (2008). Identification of SNP markers for common CNV regions and association analysis of risk of subarachnoid aneurysmal hemorrhage in Japanese population. Biochem Biophys Res Commun.

[47] Bae JS, Cheong HS, Park BL (2010). Genome-wide association analysis of copy number variations in subarachnoid aneurysmal hemorrhage. J Hum Genet.

